# FZD7: A potential biomarker for endometriosis

**DOI:** 10.1097/MD.0000000000035406

**Published:** 2023-10-06

**Authors:** Suwei Lan, Zhengmao Zhang, Qing Li

**Affiliations:** a Hebei Medical University, Hebei, China; b Affiliated Hospital of Chengde Medical University, Chengde, Hebei, China; c Department of Gynecology, The Fourth Hospital, Hebei Medical University, Shijiazhuang, China; d Chengde Medical University, Chengde, Hebei, China.

**Keywords:** bioinformatics, endometriosis, ferroptosis, FZD7

## Abstract

**Background:**

Endometriosis is a chronic inflammatory, benign disorder that often co-occurs with adenomyosis and/or leiomyoma. The overall incidence of endometriosis in reproductive period women was nearly 10%. However, the exact mechanisms of endometriosis-associated pathogenesis are still unknown.

**Methods:**

In this study, we aimed to investigate whether Frizzled-7 (FZD7) would effectively promote the development of endometriosis. The microarray-based data analysis was performed to screen endometriosis-related differentially expressed genes. This process uncovered specific hub genes, and the nexus of vital genes and ferroptosis-related genes were pinpointed. Then, we collected human endometrial and endometriotic tissues from patients with endometriosis of the ovary (n = 39) and control patients without endometriosis (n = 10, who underwent hysterectomy for uterine fibroids) to compare the expression of FZD7.

**Results:**

These findings indicated that the expression of FZD7 was high compared with normal endometrium, and FZD7 may promote the progression of endometriosis.

**Conclusion:**

FZD7 may serve as a potential therapeutic target for endometriosis treatment.

## 1. Introduction

Endometriosis is a benign gynecological disease in which endometrial glands and stroma are found outside the uterus.^[1]^ Consequently, it is a hormone-dependent disorder associated with chronic pelvic pain and infertility.^[2]^ The most common location of endometriosis is the ovaries.^[3]^ However, endometriosis carries a risk of malignancy. The risk of direct malignant transformation of ovarian endometriosis has been estimated at 0.7% to 1.6% over an average of 8 years.^[4]^ Endometriosis is a significant problem in reproductive women’s health, dramatically affecting the quality of life.^[5]^ Unfortunately, the understanding of the pathogenesis of endometriosis is relatively poor. Currently, the thinking is that the underlying cause is retrograde menstruation leading to the implantation of exfoliated endometrium in the pelvic.^[6]^ Notably, it has been indicated that gene therapy holds significant promise for treating endometriosis in patients who currently have minimal treatment options.^[7]^

Ferroptosis is a non-apoptotic form of cell death caused by iron ion-dependent, excessive accumulation of intracellular lipid peroxide and reactive oxygen species due to the peroxidative damage of cell membrane phospholipids.^[8]^ Dixon et al^[9]^ initially defined this pattern of cell death as “ferroptosis”. Recently, ferroptosis has been found to play an important role in breast cancer^[10]^ and gastric cancer.^[11]^ At the same time, it has also been verified to play biological roles in many nonneoplastic diseases, For example, Alzheimer^[12]^ and cardiovascular disease.^[13]^ The GPX4-GSH defense system is the most classic ferroptosis antioxidant defense system.^[14]^ Furthermore, recent studies have confirmed that Frizzled-7 (FZD7) could promote ferroptosis through the β-catenin-TP63-GPX4 pathway in ovarian cancer.^[15]^

FZD7, a member of the Frizzled (FZD) family of transmembrane proteins, is a Wnt receptor that can activate the canonical and/or the noncanonical Wnt signaling pathways.^[16]^ Additionally, it has been demonstrated that FZD7 plays a vital role in immature porcine Sertoli cell proliferation.^[17]^ Many cancer types also display up-regulation of FZD7, associated with cancer cell proliferation, and invasiveness, suggesting that FZD7 is important for driving cancer growth.^[18]^ For example, the increased expression of FZD7 was associated with distant organ metastasis, advanced clinical stages, and poor clinical prognosis in pancreatic cancer.^[19]^

In this study, bioinformatic screening for FZD7 was highly expressed in endometriotic tissues, and the first immunohistochemical determination of FZD7 was performed in human tissues. Our results showed that the FZD7 expression level was significantly higher in ectopic endometrial tissues than in normal endometrial tissues. Furthermore, it positively correlated with the size of ovarian endometriotic cyst, but not with stage and CA125 level in serum.

## 2. Materials and methods

### 2.1. Tissue sample collection

Normal endometrial tissues from patients (n = 10) who had a hysterectomy for uterine fibroids and ectopic endometriosis tissues (n = 39) in the ovary were collected in the Affiliated Hospital of Chengde Medical University(Chengde, China) between January 2022 and May 2023. All patients provided formal written consent to use their tissues and

clinical data in this study. Additionally, the study protocol was approved by the Institutional Research Ethics Committee of the Affiliated Hospital of Chengde Medical University(Chengde, China).

### 2.2. Data acquisition

Messenger RNA (mRNA) profiling datasets were extracted using the gene expression omnibus database (https://www.ncbi.nlm.nih.gov/gds) to compare RNA expression between endometriosis and normal uterine endometrium tissues. RNA profiling using array data was identified with ID numbers GSE11691 and GSE25628. Additionally, the platform for the GSE11691 array profiling was GPL96 ([HG-U133A] Affymetrix Human Genome U133A Array). Paired samples of eutopic (GSM296875–GSM296883) and ectopic (GSM296884–GSM296992) endometrium from 9 individual women were collected, and the transcript profiles were compared.

The GSE25628 profiling array platform utilized the GPL571 [HG-U133A_2] Affymetrix Human Genome U133A 2.0 Array, which contained 7 ectopic endometrium (GSM629719, GSM629721, GSM629723, GSM629726, GSM629728, GSM629730, GSM629732) and 6 eutopic endometrium (GSM629722, GSM629724, GSM629725, GSM629727, GSM629727, GSM629735)tissues. We downloaded the genes related to ferroptosis in the ferroptosis database (FerrDb; http://zhounan.org/ferrdb/current/), which contained an exhaustive list of ferroptosis-related genes.^[20]^ Furthermore, overlapping genes were performed to obtain the target and ferroptosis-related genes.

### 2.3. Preprocessing of data and analysis of differential expressions

Upon retrieving the processed matrix of sequencing expressions, we utilized the Limma package 6 to evaluate expression discrepancies between samples. Consequently, 2 volcano plots were generated. Genes presenting incomplete annotation details, exceptionally low expression, or duplications were excluded from the analysis. The differential messenger RNAs were screened under the conditions of FDR < 0.05, logFC > |2.0|. Moreso, we gathered overlapping target genes and conducted an enrichment analysis concerning signal pathways. Gene ontology (GO) analyses were performed via the R language cluster Profiler package (3.14.3).^[21]^

### 2.4. Protein–protein interaction (PPI) network construction

The STRING database (https://string-db.org/) was used to forecast PPI networks, facilitating downstream protein interaction predictions. Importantly, we aimed to intersect chosen target genes with ferroptosis-related proteins. The Venn diagram was constructed using the R language ggplot2 package.

### 2.5. Tissue collection and immunohistochemistry (IHC)

Thirty-nine deidentified ovary tissues with endometriosis and 10 normal endometrium tissues were collected at the Affiliated Hospital of Chengde Medical University. These tissues were processed fresh from patients who provided written informed consent. The tissues of endometriosis and normal endometrium were subjected to the detection of FZD7 expression using the IHC staining method. Samples were fixed with 4% formaldehyde, embedded with paraffin, cut into 4 μm serial sections, and placed in an incubator at 60°C for 1 hour. After deparaffinization and rehydrating the paraffin sections, they were placed in a repair box filled with citric acid antigen retrieval buffer (pH 6.0; 250 milliliters), underwent hyperthermia for 4 minutes, then microwaved on medium-low heat for 20 minutes, and cooled at room temperature for antigen retrieval. Next, sections were placed in 3% hydrogen peroxide and incubated at room temperature for 25 minutes to block endogenous peroxidase activity. This was followed by serum blocking with 3% BSA (Servicebio G5001, Wuhan, China) for 30 minutes at room temperature. antihuman IgM rabbit monoclonal antibody (1:1000 dilution; SP-9000, Maixin) was incubated overnight at 4°C. The sample was washed with PBS 3 times for 3 minutes each, followed by incubation with a secondary antibody at room temperature for 30 minutes, followed by a PBS wash 3 times for 3 minutes each. DAB kit (ZLI-9018, Zhongshan Jinqiao) coloration was utilized for these samples. The samples were washed with distilled water twice for 1minutes, hematoxylin counterstained for 1 minutes, washed with tap water for 2 minutes, sealed, and placed in a fume hood to dry. The pathological sections of different visual fields were observed under the microscope (CX43, OLYMPUS) and photographed at different multiples to analyze the pathological results.

### 2.6. Statistical methods

The R language Limma package was adopted to screen differential genes, with selection criteria being up-regulation or down-regulation with a difference multiplier > 2.0 and *P* < .05. Analyses of GO considered *P* < .05, *q* value < .2 as statistically significant. Correlation tests were performed using the Spearman correlation.

## 3. Results

### 3.1. Differentially expressed gene analysis

**Figure 1.**
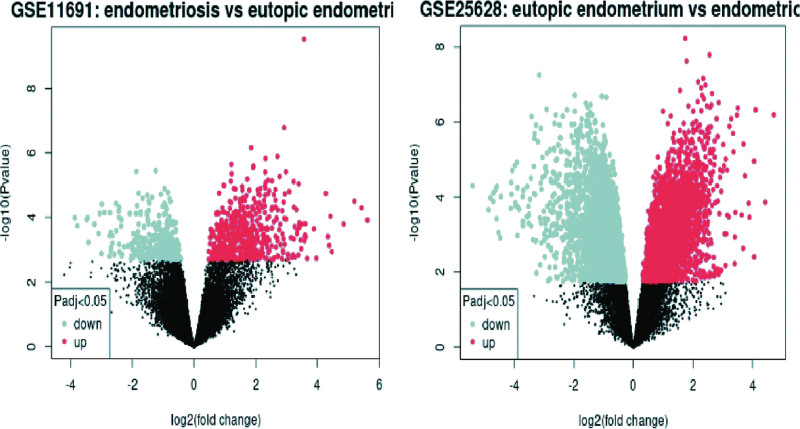
Volcano maps of DEGs in GSE11691 and GSE25628. DEGs = differentially expressed genes.

**Figure 2.**
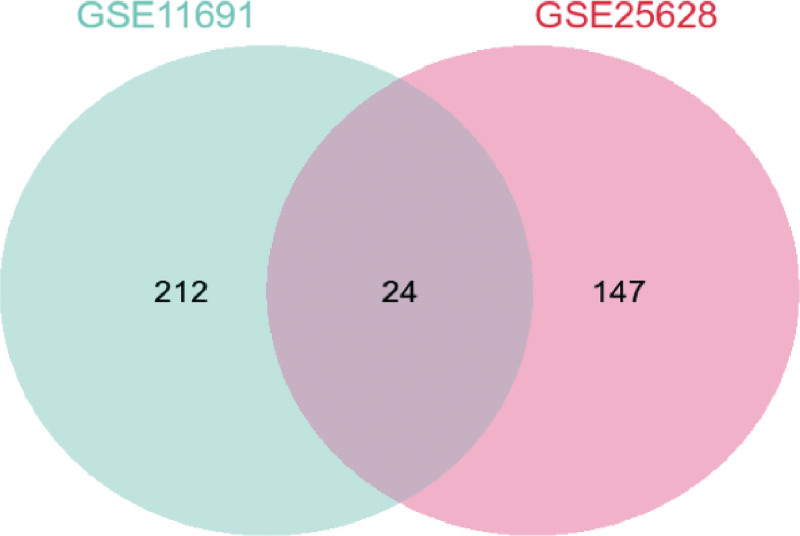
Venn diagram of screened DEGs from the 2 GEO datasets. DEGs = differentially expressed genes, GEO = gene expression omnibus.

Through comparison of the endometriosis tissue group with the normal endometrium group in GSE11691 and GS25628, differential genes volcano maps in GSE11691 and GSE25628 were created (Fig. 1). About 24 different expression genes were screened (|logFC|>2, *P* < .05) in these gene expression omnibus datasets (Fig. 2).

**Figure 3.**
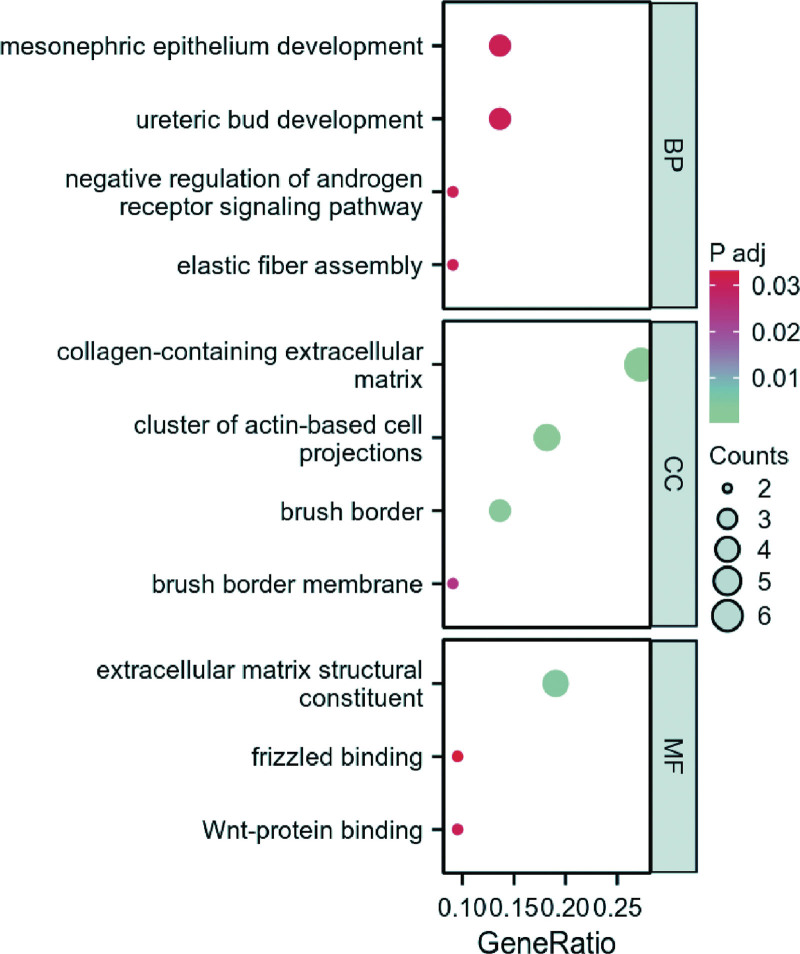
GO analyses results of DEGs. DEGs = differentially expressed genes, GO = gene ontology.

We gathered overlapping target genes and conducted an enrichment analysis concerning signal pathways. GO analyses were performed on genes via the R language cluster Profiler package. Our results indicated the involvement of mesonephric epithelium development, collagen-containing extracellular matrix, and extracellular matrix structural constituent, as shown in Figure 3. Notably, the Kyoto encyclopedia of genes and genomes enrichment analysis produced no obvious pathway.

### 3.2. PPI network construction

**Figure 4.**
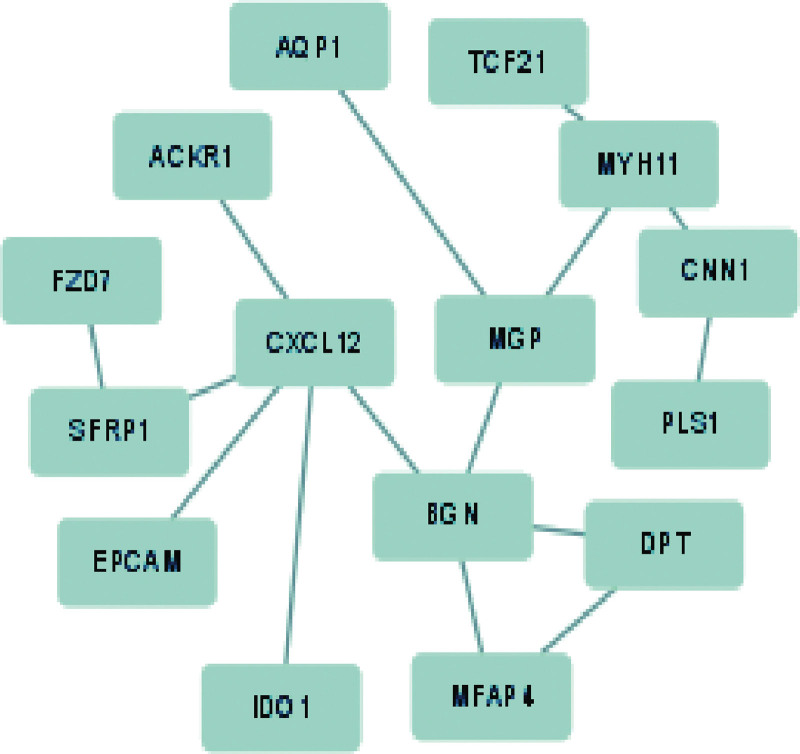
The top 15 genes.

The 24 target genes were imported into STRING datasets. The results were then entered into Cytoscape to establish a network map of target genes. The top 15 genes were designated as hub genes, as illustrated in Figure 4. From the network, the most closely connected was FZD7-SFRP1.

### 3.3. Intersection of target genes and ferroptosis-related genes

**Figure 5.**
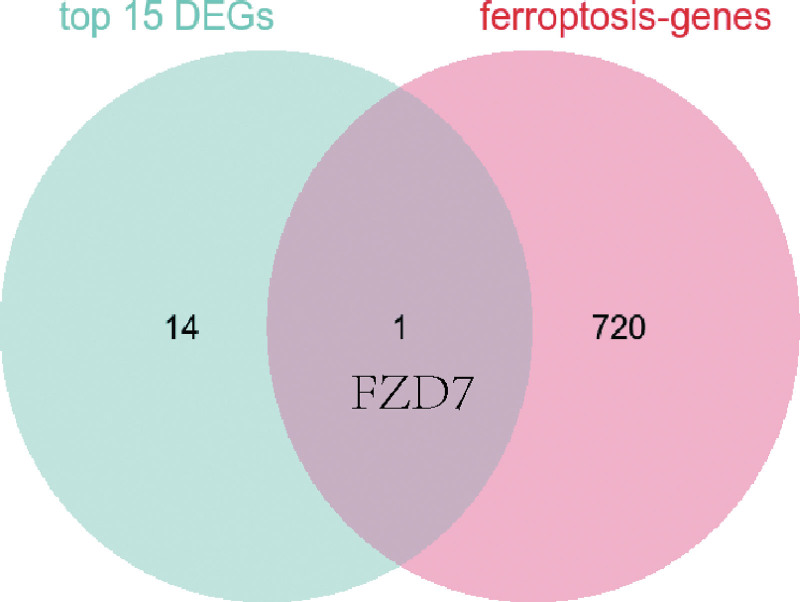
The overlapping genes.

The top 15 overlapping genes (including FZD7, SFRP1, EPCAM, IDO1, MFAP4, DPT, PLS1, CNN1, MYH11, TCF21, AQP1, ACKR1, CXCL12, MGP, and BGN) were performed to obtain the target genes and ferroptosis-related, as shown in Figure 5. Then, only 1 gene (FZD7) was screened in the Venn diagram.

### 3.4. FZD7 expression profiles s in normal endometrium and ectopic endometrium in ovarian tissues

**Figure 6.**
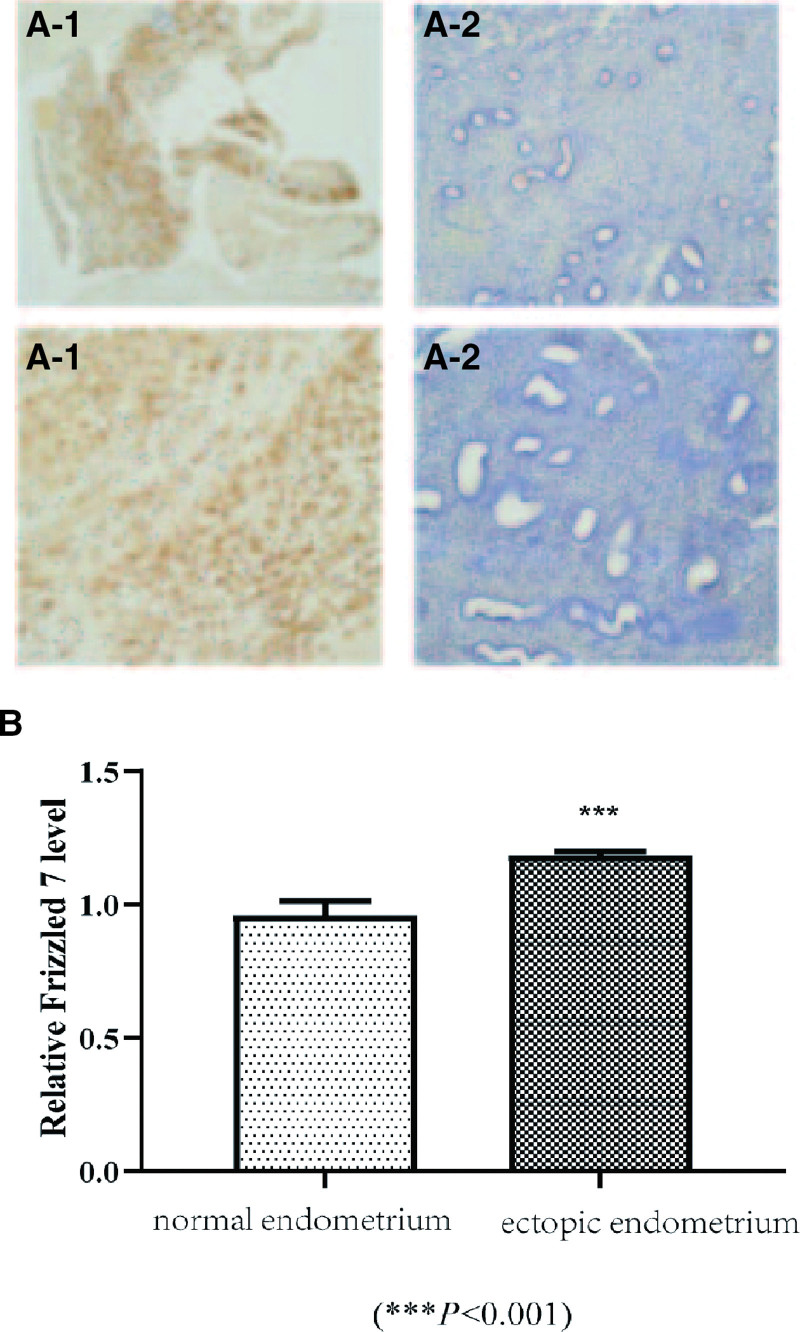
Expression of FZD7 in endometrium tissues. (A) Representative images of immunohistochemistry showing FZD7 expression in ectopic endometrium tissues; Representative images of immunohistochemistry showing FZD7 expression in normal endometrium tissues. (B) Relative FZD7 expression in two groups. FZD7 = Frizzled-7.

IHC staining showed that FZD7 was enriched in the cytoplasm and the cell membrane in ectopic and eutopic endometrium. As shown in Figure 6A, the immunohistochemical score showed that the level of the FZD7 protein in ectopic endometrium tissues was significantly higher than in the eutopic endometrium (*P* < .001, Fig. 6B).

### 3.5. The relationship between the expression level of FZD7 and the clinicopathologic characteristics of patients in endometriosis tissues

According to the mean relative expression of FZD7 (91.057), the endometriosis patients were divided into low FZD 7 expression and high FZD 7 expression groups. A comparison of the clinicopathologic features of the 2 groups of endometriosis illustrated that FZD 7 expression was related to endometriotic cyst size and not related to the endometriosis stage, age, and serum CA125 level (Table 1).

**Table 1:** The relationship between the expression of FZD7 and clinicopathological characteristics of endometriosis patients.

	n	FZD7 level		*P* value
		Low expression	High expression	
Stags				.333
II	4	3 (7.7%)	1 (2.6%)	
III	16	7 (17.9%)	9 (23.1%)	
IV	19	6 (5.4%)	13 (33.3%)	
Age (yr)				.523
≥40	19	9 (23.1%)	10 (25.6%)	
<40	20	7 (17.9%)	13 (33.3%)	
CA125(U/mL)				.523
≥35	19	9 (23.1%)	10 (25.6%)	
<35	20	7 (17.9%)	13 (33.3%)	
Cyst sile (cm)				.004
≥6	31	9 (23.1%)	22 (56.4%)	
<6	8	7 (17.9%)	1 (2.6%)	

FZD7 = Frizzled-7.

## 4. Discussion

It is well known that endometriosis is a chronic disease that has affected about 5% to 10% of reproductive-age women. The most common ectopic site is the ovary.^[22]^ The guidelines recommend surgery for pelvic pain that fails medical treatment and endometriosis forming a pelvic mass > 4 cm cure.^[23]^ However, its high risk of recurrence^[24]^ and the long duration of malginancy^[25]^ still troubles clinicians regarding the treatment of endometriosis. Thus, understanding the molecular mechanisms of endometriosis of biomarkers and therapeutic targets is crucial.

Ferroptosis, as a new way of programmed cell death, has been paid more attention in nontumor-related research in recent years, mainly caused by the accumulation of lipid peroxidation products, which will lead to cell death after reaching a lethal dose.^[26]^ Additionally, recent studies have shown that FZD7 could inhibit ferroptosis in ovarian cancer through the β-catenin/TP63/GPX4 pathway.^[15]^ Another study verified that BCL6 facilitated ferroptosis via the FZD7/β-catenin/TP63/GPX4 pathway in gastric cancer.^[14]^ As one of the ferroptosis-related genes, we speculated that the mechanism of FZD 7 in the disease is related to ferroptosis.

FZD proteins are a class of Wnt recipients with 7-fold transmembrane molecular structures Body family, with 10 family members in mammals (FZD 1 to FZD 10).^[27]^ FZD 7 is one of the FZD receptor family members and was first cloned and identified in 1998.^[28]^ Studies have shown that FZD7 is the only member of the FZD receptor family that activates each branch of the Wnt pathway (Wnt/β -catenin pathway, Wnt/ PCP pathway, and Wnt/ Ca 2 + pathway).^[29]^ FZD7 plays an important role in regulating cell polarity, embryonic development, other life activities, and the occurrence and development of tumors.^[30]^ In addition, FZD 7 also plays a role in the occurrence and development of many nonneoplastic diseases.^[17,31]^ Hui Zhu et al^[32]^ first reported that FZD7 was highly expressed in mice’s endometriotic tissues. Our study first demonstrated that FZD7 was highly expressed in ectopic endometrial tissues compared to normal human endometrial. Further Spearman analysis revealed that it was significantly related to ectopic cyst size, but there was no correlation with clinical stage, serum CA125 levels, and age.

PPI network analysis revealed that FZD 7 is closely associated with SFRP 1. A study reported that compared with the eutopic tissues, SFRP1 messenger RNAs is significantly higher in ectopic tissues.^[33]^ Furthermore, the relationship between FZD 7 and SFRP 1 was further confirmed by experimental studies.

In summary, we clarified that the expression of FZD7 was higher in ectopic endometrium than in eutopic endometrium and may be a potential therapeutic target for the prognostic marker for endometriosis still need further study.

## Acknowledgments

The authors thank AiMi Academic Services (www.aimieditor.com) for English language editing and review services.

## Author contributions

**Conceptualization:** Suwei Lan, Zhengmao Zhang.

**Data curation:** Suwei Lan, Qing Li.

**Formal analysis:** Suwei Lan.

**Software:** Suwei Lan, Qing Li.

**Validation:** Suwei Lan.

**Visualization:** Suwei Lan.

**Writing – original draft:** Suwei Lan, Zhengmao Zhang.

**Writing – review & editing:** Suwei Lan.
